# Understanding intercultural transitions of medical students

**DOI:** 10.5116/ijme.54e7.b57f

**Published:** 2015-02-28

**Authors:** Aneta L. Hayes, Nasser Mansour, Ros Fisher

**Affiliations:** 1 School of Public Policy and Professional Practice, Keele University, UK; 2 Graduate School of Education, Exeter University, UK

**Keywords:** t ransnational medical education, transition, communities of practice, identity, agency

## Abstract

**Objectives:**

The aim of this research was to explore the transition of medical students to an international branch campus of a medical university established in Bahrain.

**Methods:**

In order to gain insights into this transition, we explored two culturally diverse systems of learning of the university and the local schools in Bahrain, using Communities of Practice as a lens for understanding transitions. Focus groups were conducted with secondary school teachers and first year medical students. Additionally, semi-structured interviews were conducted with university lecturers.

**Results:**

The findings suggest that, while Communities of Practice have been influential in contextualising transitions to university, this model does not seem to help us to fully understand intercultural transitions to the case-study university.

**Conclusions:**

The research emphasises that more attention should be given to learner individual agency within this theory as a framework for understanding transitions. It also challenges approaches within medical education that attempt to standardise systems of learning through acquisition of established practices.

## Introduction

The research presented in this paper describes a transition of students from Bahraini mainstream secondary schools to a medical university which has been ‘transplanted’ with its pedagogical values and context of teaching practice to the culture of Bahrain. The fact that the teaching practices of the university have been transplanted to the context of the medical students is important for increasing our understanding of how students learn through models such as Communities of Practice (CoP). Therefore, through exploring this transition, the aim of this paper is to critically evaluate the usefulness of CoP as a lens for understanding intercultural transitions, which has practical implications for medical educators interested in enhancing the student experience through practice. To contextualise the focus of this study, we first provide background literature that helped us rationalise our research and definitions of key concepts that we used. We then explain how this had led us to focus on CoP and we provide essential information about this model of learning.

### Context of the study and rationale 

While the older models of globalising medical education have been based on sending home students to medical schools abroad, more recently, this has been replaced with establishing international branch campuses in students’ own countries.^1^ Particularly the Middle East has seen a growth in international branch campuses, with the first medical school, Weill Cornell, being established in Qatar. The college to which the transition is considered in this study is also an international branch campus which has been established in Bahrain through curriculum transfer from the parent institution. The undergraduate programme in the college is built on an outcomes-based model which is primarily lecture-based in the early years and supplemented by a number of tutorial and laboratory sessions.

According to Rosenthal,^2^ models of higher education based on curriculum transfer may pose obstacles to transition for locally educated students.

Those students are usually taught in their native languages prior to joining university, which makes it difficult for them to   acquire large amounts of material from high-content didactic lectures that are widely applied in outcomes-based medical programmes.

Rosenthal^2^ claims that, often, when English language minority students transition to foreign higher education (HE) contexts, the focus on language becomes dominant and the scientific knowledge delivered in lectures becomes lost. The transitional experience therefore becomes dominated by the level of the English language and conceptions of learning that students had developed before beginning university.  In addition, the socio-cultural factors connected with transitioning between two cultures of learning may make transitions negative.^1^ Research, therefore, focuses on exploring these factors, looking into conceptions of learning foreign students bring with them from their cultures^3^ and the level of difficulty language barriers might cause.^2^ We too focus on these aspects of students transition, trying to understand how the socio-cultural influences on previous education in science and the language proficiency of medical students affect the transitional experience of learners in Bahrain.

Gu, Schweisfurth and Day^3^ conducted a similar study to the one described in this paper in the context of UK universities whose aim was to explore the complexities of international students’ transitional experiences. Their findings suggested that a combination of organisational, social, cultural and personal factors influenced students’ adaptation to and identity change in the new higher education HE contexts. While social and cultural development was an important element of transition in Gu, Schewisfurth and Day’s^3^ study, where students demonstrated high levels of adaptation to a new culture, factors connected with acculturation were considered less important in the context of the present research due to the location of the medical university in Bahrain. On the other hand, we decided to focus on learning and the educational environments of the schools and the medical university. This also influenced the definition of transition that we adopted, which arose from our view that transitions of medical students in transnational contexts have to be transcultural – meaning, they have  to encompass how two cultures of learning are interacting.^4^ Hence our focus on teacher, student and lecturer perspectives.

In addition to this view, we also concur with Gu, Schweisfurth and Day’s^3^ argument that transcultural transitions encompass cross-cultural transitions because they involve crossing boundaries created by differences between the two cultures. Research into these types of transitions therefore has to focus on exploring the two cultures,^5^ which was achieved  in this study through comparing the  perspectives on the role of school culture in Bahrain and the medical university from different groups of secondary teachers in Bahrain, first-year university students and university lecturers.

### Conceptualisations of transition

Transition is often described in literature as a form of change.^4^^,^^6^^,^^7^ In educational contexts, it has been noted that this change may take place on a more personal level, for example, in terms of new beliefs or developmental growth.^3^ It may also involve a physical move from one place to another, such as going from primary to secondary school or leaving a home country to study at the university abroad.^8^ Lam and Pollard ^9^ differentiate between two types of educational transitions and describe them as changes of an institutional setting and as changes of a personal context. We believe that the former one is particularly relevant in transnational contexts because when a medical HE institution arrives in the country of students, it is usually the culture of that institution that interacts with the culture of students. Additionally, in situations when international branch campuses are established in the countries of students, little change in the personal context of the medical students takes place because they are still able to interact within their own national cultures. We also believe that the culture of medical students that interacts the most with the new institutional culture is that of their schools because of the absence of factors related to social adjustment, incongruence and isolation that are common in transitions of students who choose to study abroad.^10^

When discussing transitions to higher education in terms of adjustment, Tinto^10^ proposes that students are unable to adapt to a new environment because the degree of separation from their past life and successful transition to a new life is too high. Cano^11^ supports this view on transitions by concluding that separation from parents and fear of loss of cultural identity increased the levels of sociological distress of Latina students in a predominantly white American university.  Despite illuminating, these findings cannot however be fully transferred to the context of the present study.

Firstly, due to the fact that the university is located in Bahrain, the local medical students are not forced to separate from their old lives and are not required to move away from home. Therefore, their cultural identity remains preserved and can be practised on daily basis. Moreover, these students still have the opportunity to mix within their culture, as the majority of students studying at the university are Bahraini or from other Gulf Cooperation Council (GCC) countries. Additionally, Lopez^12^ explains that when transition occurs in the context where the majority of students are former school peers, or at least know that they come from the same educational background, students tend to adjust better because they feel that understanding the common background creates an additional sense of security. Being part of the Bahraini group of learners therefore does not seem to require the medical students to undergo any cultural changes and allows them to build a social orientation that makes their Bahraini peers a source of support for better integration. Therefore, the perspective on transition that has been identified as the most suitable for this study is related to intellectual difficulty and academic capability of medical students beginning HE.

Considering that the transition in this research involves crossing boundaries between two cultures of learning and that it is focused on factors that are believed to cause academic difficulty, we define transition as changes in identity and agency students need to undergo in order to move through the educational outcomes of the first year programme at the university.^6^ We adopt a socio-cultural perspective on transitions because of the interaction of  two distinct cultures in our study. Hence, the focus on identity and agency which will be explained below.  Finally, because we are interested in the academic difficulty connected with learning science in English, we explore educational practices of English language and science teaching in Bahrain and their role in preparing students for a study of medicine in a linguistically and culturally diverse context.

### Brief literature review and conceptualisations of identity and agency in this study

Research on transitions from secondary to tertiary education, especially in the context of language and culture change, often addresses the level of opportunity given to students by their school contexts to develop adequate tertiary English language and disciplinary skills.^13^ Pedagogy of secondary contexts is generally believed to be responsible for equipping learners with attributes and characteristics which are then packaged by the students in a way that is useful in the new context of the university.^13^^,^^14^  These attributes and their strategic use in higher education are understood in this study as identity and agency.^15^

The definition of transition adopted in this study refers to the ‘movement’ through the educational outcomes of the university which is expected to result in learners’ shifts in identity and agency.^6^ According to this definition, these shifts have to take place in order for students to progress through the educational system of the university, which makes transitions socially regulated and dependent on the institutional settings.^6^ This means that when learners undergo this type of transition, they need to discover the best ways of responding to institutional pathways and normative expectations, utilising their attributes as learners. Thus, identity is defined in this study as distinctive characteristics of individuals which are shared by all members of a particular social group and which are changing as learners interact in the new social context.^15^ Agency is referred to here as the ways in which people make choices to respond to situations based on the opportunities given to them by those distinctive characteristics.^15^

Changes in identity, therefore, will be explored by understanding the extent to which being a learner in school transfers to being a learner at university, and changes in agency will be examined through exploring   whether coping strategies in school and past actions can also assist students in their learning at third level. We hold the view that identity and agency are inextricably interconnected because actions taken can be built or constrained by specific learner characteristics.^6^

Due to the possible constraints caused by learner characteristics, many universities offer orientation programmes which aim to show students how to effectively ‘package’ the resources that they bring with them to respond to the educational practice in HE.^16^^,^^17^^,^^18^  The university where this study took place is no exception and a specific orientation programme which teaches students about learning from lectures, participating in tutorials, forming study groups and thinking critically in English has been designed.

The rationale behind this programme is that the sooner the students are informed about the pedagogical practice of the university, the sooner they will follow this practice and their transition to tertiary education will be enhanced.^19^ This rationale was developed based on  the socio-cultural model of Communities of Practice (CoP)^20^  as a framework for understanding transitions to HE21 which advocates knowledge-sharing and development of desired skills through apprenticeship and explicit, guided teaching by experienced members of the university community. CoP propose that when there are too many differences between the practices of two communities of practice, transitions may be affected in a negative way.^21^ Consequently, we aimed in this paper to evaluate how this model helps us understand the experiences of medical students from the specific Bahraini context at a European university, where differences are likely to exist. We also aimed to critique if CoP can be a useful lens for understanding the transition to the case-study medical university.

### COP as a model for understanding transitions

In CoP, the focus is on practice and identity, as well as how these two concepts mediate transitions between two separate communities.^20^ The two communities in this study were national schools in Bahrain and an international medical university. According to Wenger, ^20^ to move between these communities, individuals need to negotiate their identities in a way that will provide access to a new community and its practice. Through these negotiations, learning takes place and in CoP, learning means the shift of identity in the face of participation in and adaptation of practice.^20^

Wenger^20^ refers to the process of identity negotiation as forming trajectories and states that we constantly need to renegotiate who we are in order to cross boundaries across communities. . This means following and learning from the already established members of the new community and developing characteristics that guarantee success in this community. By right, some trajectories never lead to full participation in a new community but it is believed that the newcomers joining this community always have intentions of becoming full participants in its practice.^20^ These newcomers are legitimate peripheral participants because they have experience, knowledge and characteristics that set out how quickly they will be able to move from the periphery of the new community to the centre.^20^ It was therefore important for us to investigate which  trajectories existed for Bahraini students entering the new community of the medical university and whether these trajectories allowed them to achieve full participation. This links in very well with the purpose of orientation programmes offered by universities whereby already established university members are expected to teach novice students about the practice of the new environment. CoP, therefore, have become a useful framework for understanding transitions because novices entering university have often  been found to reconsider who they are as learners in order to ‘fit’ with the way a community of practice does or views things.^21^^,^^22^

Identity and practice, therefore, often become the focus of research which uses CoP for understanding transitions. Tobbell, O’Donnell and Zammit,^23^ for instance, found that students’ former learning identities played a role in their transition in that they suggested links between learning and self-efficacy characteristics formed in previous communities of practice. Tobbell, O’Donnell and Zammit’s^23^ students reported challenges in negotiating their academic identity in light of previous educational practices, especially the need for independent study, which was the practice that necessitated identity change in the community of the university in their study.

In our study, we have also used CoP as a lens for looking at the transition to the medical university with the view of developing an understanding of what changes in identity and agency our medical students undergo.  In order to understand the learning identities of our students and the impact of previous educational practice on their transition, we had to investigate the socio-cultural framework of our students’ education and explore their present and past learning experiences. We therefore aimed to explore different understandings of the medical students’ transition into the first year of university expressed through the views of their secondary teachers, their university lecturers and students themselves. We posed the following research questions: ‘How do secondary teachers, medical students and university lecturers perceive the role of school practices of English language and science education in students’ identity and agency change as learners transition to university?’ and ‘What does this mean for CoP as a lens for understanding transitions?’

## Methods

### Study design

To answer our research questions, we adopted an interpretive case study methodology. The case in this study was Bahraini students in a medical university and adopting this methodology allowed us to provide an in-depth account of events, relationships, experiences and practices that shaped students’ identity.^24^ While we agree that the subjectivity of case studies might be a limitation, we felt it was more important for the objectives of this study to adopt this methodology because it gave us real insights into the practices that the medical students deploy as agentic learners. The study was approved by the Research Ethics Committee at the university where it took place as well as by the Ministry of Education in Bahrain.

### Participants

The study participants consisted of 4 groups: (1) medical students from the university where the study took place, (2) English language teachers in secondary schools, (3) science teachers in secondary schools and (4) university lecturers. We chose to interview medical students because gaining insights into their experiences was invaluable for us to understand their transition. We also chose secondary English and science teachers because these two aspects of the new medical students’ secondary education were found by the university to have the greatest impact on their transition.^25^ We finally interviewed university lecturers to gain their perspectives on the transition, which added an additional voice of people who had a direct impact on student transition in this case.^26^

### Sample size

35 medical students out of 41 eligible to participate (please see section ‘Sampling Method’ regarding the sampling criteria) took part in the first round of focus groups, only 9 of those took part in the second round. We managed to interview 60 English language teachers and 22 science teachers. All university lecturers (n=6) who teach the first year programme at the medical university were interviewed in the study.

### Sampling method

1. Student participants were selected by means of purposive sampling strategies and we selected only Bahraini students who (1) were first year university students studying medicine, (2) graduated from the Bahraini mainstream state education system with national qualifications Tawjihya, (3) studied sciences in Arabic and (4) spoke Arabic as their first language. All participants were 18 years old. Student participants were coded according to those who attended the English programme at the university (ELC) and those who were exempt from this programme (Non-ELC) (All medical students are required to present their International English Language Testing System (IELTS) scores on entry to the university. If students present scores of IELTS 6.5, they are exempt from the English programmes). 

2. English language teachers were selected randomly from all national schools in Bahrain. These teachers were employed full time. Their teaching experience in Bahraini schools ranged from 2-30 years and they were all educated to a degree level. Their responses were coded ‘ETeacher’. Numbers and letters were additionally used to identify teachers in individual schools, e.g. an English teacher who was given number 1 in school A was coded as ‘ETeacher1A’.

3. Science teachers were also selected randomly. The science teachers were also working full time, with teaching experience in Bahrain ranging from 5 - 20 years. They all had degrees in science education. Their responses were coded ‘STeacher’. Similarly to English teachers, science teachers were given letters and numbers as codes and, for example, a response from a science teacher 2 in school C was coded ‘STeacher2C’.

4. Finally, all 6 lecturers who deliver the courses in the first year at the medical university participated in the study. They were all educated to a PhD level and their teaching experience ranged from 8-50 years. The responses from lecturers were coded Faculty 1 – Faculty 6.

### Data collection

Focus groups were chosen to collect data from school teachers and the medical students. University lecturers were interviewed individually. Teachers in each school were interviewed once. Two rounds of interviews were conducted with the medical students.

The questions for English and science teachers sought to explore English and science teaching practices (e.g. ‘Can you explain how you teach English?’, ‘Can you talk about science programmes in Bahraini schools?’) and teachers’ perspectives on the role English and science teaching pedagogy may play in students’ adjustment to higher education (e.g. ‘What do you think should be done to respond to students’ English language needs at third level?’, ‘What is good/ bad about science teaching in terms of students’ future careers at university?’). The teachers were also asked to talk about the context of schools in which their teaching takes place (e.g. ‘What determines the way you teach English?’, ‘What guides teaching science in Bahraini schools?’).

In the first round of the focus groups, the medical students were asked about their experiences with learning at university, as well as how this experience compared with learning in school. The questions focused on the students’ feelings in terms of what they found easy and difficult at university and what facilitated or hindered their learning (e.g. ‘How do you feel now about studying at university?’). They were also asked to elaborate on the practices of English and science teaching in school and their relation to the transition (e.g. ‘What was learning English like at school? Can you compare it to what is needed now at university?’). Views on the use of specific learning strategies were exchanged too in order to establish their importance for success in higher education (e.g. ‘Were the tasks at school similar to what you’ve done this semester? What skills did they involve?’). The second round of the focus groups concentrated on discussing the role of school culture in students’ transition (e.g. What aspects of school culture play a role in making a transition to a university like this one?’). The medical students were also asked whether they had undergone any identity changes (e.g. Do you feel you have undergone any changes in order to be able to study at this university?’)

Semi-structured individual interviews were conducted with university lecturers. The questions asked intended to gather information on their perspectives of what facilitates or hinders the transition of local students to the medical university (e.g. ‘What in your view are the biggest problems with learning FY students face?’). Hence, each faculty member was asked to discuss their individual expectations in terms of the English language and science, relating to the level of language and science knowledge demand in their subjects (e.g. ‘What are your expectations in terms of English skills students should develop to achieve success in your subjects?’)

### Procedure

The focus groups and the semi-structured interviews were audio recorded. All participants were interviewed by the first author. The teachers were visited in their schools and the students and their lecturers were interviewed on the university premises. All interviews with English teachers were conducted in English but, due to the fact that these teachers were native speakers of Arabic, the interviews protocol and questions were back translated from English into Arabic. The interview protocol and questions for the science teachers were also translated into Arabic. Additionally, the science teachers were allowed to speak in Arabic during the focus groups due to poor proficiency in English. Those parts of the focus groups which were articulated in Arabic were translated for analysis using the back-translation method. An interpreter was present during all focus groups with the science teachers to translate parts of conversations that took place in Arabic. Each interviewee was given a copy of the interview protocol with questions written in both languages.

The medical students were also given copies of interview questions and the protocol in Arabic. The university lecturers received copies of the protocol and interview questions in English because they were all native speakers of English.

On average, the sessions lasted between 40 - 60 minutes. All data were sent back to interviewees for participant validation. No comments with corrections were returned.

### Data analysis

The data in this study were analysed inductively and constant comparisons between all participant groups were made to see how different interviewee groups interpret the role of school practices in teaching English and science in the medical students’ transition. The Constant Comparative Method^27^ was used to identify whether any patterns in interpretation regarding the role of practices in relation to these two subject areas existed and whether the data suggested a similar understanding of transitions to that of CoP. Constant comparisons were also used because, in interpretive research, comparing between different participant groups adds rigour to the process of analysis and develops  greater confidence in the findings through using multiple sources of evidence.^24^ All interviews were transcribed in full and segments of text were summarised with key words (referred in Table 1 as ‘examples’), developing individual codes related to practice of English language and science teaching. Interesting codes arose during data analysis in relation to the general socio-cultural context of educational practices in Bahraini schools and these were grouped under the category of Pedagogy. Table 1 below presents all codes, key words and explanations under each category. Analysis was undertaken by each author individually and the codes from each author were compared to increase the credibility of the developing themes that are discussed in the results section.

**Table 1.** Categories and Codes identified during data analysis

Category	Code^*^	Explanation/ Example
1.Science background knowledge	Standard of science education (01B) Practices and approaches to teaching science (02B)	The codes under the category of science background knowledge refer to the role of science base, approaches to study science and skills in science in transition Examples: good for university, high level of science in school, old science curriculum very detailed , high level of science helped overcome language difficulties Examples: focus on theory, learning science based on memorisation
2. The English language	Language skills (01E) Practices and approaches to teaching English (02E) Professional Language (03E) General proficiency in English (04E) Broader context of schools (05E)	This category refers to statements which concern the role of the English language in students’ transition. Examples: focus on specific skills, importance of writing, importance of grammar Examples: communicative approach, memorisation of model answers, system of awarding marks Examples: importance of medical terminology, impact of medical terminology on understanding of science Examples: Importance of language proficiency, good language users do not perform better Examples: focus on the final exam, object of teaching, emphasis on theory
3. Pedagogy	General school pedagogy/ practices (01S) Goal of education (02S)	Under this category, statements that referred to the role of broader school practices, focus of teaching and culture of schools in students’ transition were grouped. Examples: teaching not based on critical thinking, teaching based on memorisation, spoon-feeding Focus on the final exam, achieving high marks, prepare for final exam

*Codes are acronyms such as 01B, 03E or 01S. These have been developed to code the transcripts more conveniently, instead of repeating the description of codes each time they occur in the text. B stands for base in science, E stands for English and S stands for school pedagogy.

## Results

Through exploring the perspectives on transition of the secondary teachers, medical students and university faculty, four themes were identified. These themes included: (1) Inadequacy of and lack of confidence in school pedagogies, (2) The value of professional terminology, (3) Blame on local practices and (4) Confidence in old practices vs. the practice at university.

### (1)  Inadequacy of and lack of confidence in school pedagogies

All science teachers seemed to agree that due to the focus on theory in science teaching in school, Bahraini students will have to undergo a major change in their identity as learners in order to make a transition to the medical university.  They will have to understand that learning can no longer be based on memorising theoretical information. The teachers stated that building a new understanding of what learning involves might be hard for Bahraini students because in school, according to STeacher 3A,

“it is not hard, not higher thinking, there are some questions that are higher thinking (sic) and we don’t practise that with them.” (STeacher 3A)

STeacher 6B explained that this is because,

“we don’t concentrate on how to extract and how to figure out the concept from the phenomena.” (STeacher 6B)

One chemistry teacher illustrated this change by referring to the following:

*“Analysis in chemistry is missing, they will find it hard at the university because here it is only simple … There is no graphic analysis … There was some, in the old books but they deleted it … but here the part of alkenes and alkalines is simple, just their symbols, it is not in details.” *[translated quote] [STeacher3C]

Another teacher described this change by listing the skills that future medical students need to develop in order to make a transition to university and highlighted that these skills cannot be based on memorisation.

*“The first thing student must be skilled in mathematics. That is the first thing that prepares him for the university. The second thing is (sic) the proficient of physics rules and their deriving. The relations between the basic rules and the graphs (sic) Student must understand how change from this law of another law and understand how he can use of results … ... Also, there is sometimes, (sic) the student have the strategy of how to apply the rules in answering the problems in physics … .. (sic) You need not to memorise, you need to understand.” *[original quote][STeacher 2C]

The importance of similar skills in English for students’ transition was also discussed by the English teachers. Unfortunately, the interviewees were not confident that this would be easy for Bahraini students because, for example:

*If they go to the college where all the subjects are taught in English, they will have to be able to speak well to express their opinions. They will also have to have good listening comprehension to understand what they are saying. Writing should be specific to their needs, but here, (sic) like a letter or a story, I don’t think it’s relevant. So they need to connect, to make a link between the college and high school.* [ETeacher 4J]

The reason why English teachers do not teach what is required in higher education was linked by many participants to traditional school practices that encourage obedience and teacher-centred approaches. These practices were reported  not to teach the students that learning English can come through interaction because, according to ETeacher 1H, when teachers use communicative approaches that could develop skills required for university “the students think that English is easy because teachers have fun and teachers play.” (ETeacher 1H)

ETeacher 1H also explained that [Bahraini students] ‘follow teachers who are strict’ (ETeacher 1H) and cannot appreciate the benefits of opportunities to interact in English.

Additionally, the opinions of all teachers were that the emphasis on the final exam requires them to provide students with model answers. The teachers lacked confidence that ‘learning off’ the model answers that are promoted by school pedagogies develop appropriate levels of free expression in English required in higher education.

*Codes are acronyms such as 01B, 03E or 01S. These have been developed to code the transcripts more conveniently, instead of repeating the description of codes each time they occur in the text. B stands for base in science, E stands for English and S stands for school pedagogy.

“This is something that is very sad. In the writing courses, they usually give models of writing, so teachers stick to the model, students stick to the model, and we are not allowed to give them anything different. When it comes to the final exam, if you want to come up with ideas that are more creative and when the students can express themselves clearly, the newspapers will write that this is irrelevant and prevents the students’ progress. So there are a lot of complaints about teachers. If you want to give them just a little bit of free writing, this will be a problem. And the paradox is that when they explained the objectives of the curriculum it was aimed at creating creativity and free expression. This is only in theory; in practice it is completely different.” [ETeacher 4F]

Therefore, all teachers agreed that what is practised in Bahraini schools affects students’ transition in a negative way because, according to ETeacher 3B, 

“they have ready-made writing, they memorize it and when you ask them to write an independent sentence, outside that writing model, they cannot”. (ETeacher 3B)

This suggests that teachers seem to think that the identity of medical students will be challenged when they enter a linguistically diverse higher education institution. 

### (2)  The value of professional terminology

The views of science and English teachers were also similar in that they believed that the major change when students join the university will be related to the change in language. The statements by science teachers indicated that many teachers think that *“studying in Arabic and then in English is a factor in transition”* (STeacher 4C) and that *“simple medical terminology should be taught at secondary level.” *(ETeacher 4A)

The teachers seemed to imply that students’ linguistic identity might not be sufficiently developed, affecting learner agency in that lack of appropriate language resources might limit their capacity to take strategic actions. According to the theoretical framework, these resources could assist the medical students in becoming members of the new university community of practice, but according to the English teachers, they were denied to students at secondary level by inadequate teaching policies which no longer offer courses in professional terminology. ETeacher6G recalled that such courses were taught in the past and  ‘[they] were all specialised, related to their field and (sic) that they were interesting for students (sic) [and useful] for university’ (ETeacher 6G). Another teacher highlighted that these courses could be very useful for the medical students because:

Especially science students are always the best, in all schools in Bahrain, they are taking care of their studies, if we give them ‘Beginning Scientific English’ [‘Beginning Scientific English’ is the name of one of the professional terminology courses that was taught to science students in the past], like we used to in the past, (sic) that would help them with the university studies. [ETeacher 1E]

### (3)  Blame on local practices

A great sense of blame could be felt when the teachers were talking about local school practices which were developed as a result of previously mentioned learning through memorisation. According to all teachers, this style of learning leads to specific spoon-feeding pedagogies and, “they [students] want only (sic) a ready teacher to summarise for them and teach them. The good teacher (sic) who is the one who is going to give them, spoon-feed them, make it easier, simplify the subject.” [Original quote] (STeacher 2D)

ETeacher 1I reported that “[students] are used to receiving everything from the teachers” (ETeacher 1I) and ETeacher 8C added that “problems with self-study are linked to the system of education” (ETeacher 8C). This teacher also said that to ease the students transition “we don’t want the teacher to be the focus, we want to encourage the students to study (sic) for themselves, not to depend on the course of the study at school” (ETeacher 8C).

### (4)  Confidence in old practices vs. the practice of university 

Despite inadequacy, blame and lack of confidence in school policies and practices implied by the teachers, the data gathered during the focus groups with medical students suggested very high levels of confidence and empowerment.  It seemed that the medical students were undergoing the transition with an increased sense of agency because they felt confident about their background knowledge in science that they developed in school. It is therefore possible that the reasons for deploying the same study strategies at university were related to the fact that the medical students in this study were a very homogenous group. Greater variations in student voices, on the other hand, could have emerged if they had been drawn up from different schools.

For example, one medical student reported that “the science at school was so strong” (Non-ELC2 Student 1), and another one agreed that it enhanced their understanding of the lecture content and helped them overcome language barriers because “the basics of science, (sic) we have it from school and we are just relearning it now in English.” (Non-ELC1 Student 5) Another medical student indicated very clearly that knowing that s/he could rely on background knowledge facilitated the transition process: 

“What was easy was that all the material that we are taking now, we (sic) have taken in government schools, maybe even in deeper ways, not in a way we are [studying it] now, so that’s why it was very helpful. Because we know what they are talking about, we understand the whole subject”. [Non-ELC1 Student 3]

During the focus groups, it also became apparent that the confidence in memorisation strategies additionally increased the students’ sense of agency. The medical students highlighted that being able to adopt old learning strategies based on memorisation enabled them to meaningfully act on the old practices in the new setting. One medical student explained “of course that we need to understand the lectures, but it is, I think like 60% to memorise.” (ELC1 Student 3)

Another student agreed and emphasised that knowing that they could rely on memorisation gave the medical students confidence in the material and created a sense of security.

“I think memorising is a backup for understanding. When you understand something and when you, like, memorise it, you feel confident. Even when you go (sic) in the exam and you forgot the things you understood, at least you memorised it.” [ELC2 Student 2]

It seems that the medical students preferred to use what they know about learning in their transition, rather than follow the new practice given to them by the community of the university. As a result, they developed the strategy of translating lectures from English into Arabic and then back into English. One medical student explained that “some people, they translate the whole lecture from Arabic” (ELC2 Student 1).

The university lecturers at the medical university who were interviewed in this study were concerned about this strategy as, in their opinion, this way of learning might lead to the loss of information. Faculty 1 agreed that this might be an issue because “their reading skill has to be fluid, it has to be easy, and then, the expression … ..  if they are not able to logically follow through each part, they won’t understand what they get.” (Faculty 1). At the same time, all lecturers stated that this does not seem to be the problem for Bahraini students who:

(…) worked out their way of doing that and what they are doing is translating English into Arabic and back, some of the materials that we do with them is what they studied in their Arabic curriculum and they are able to transfer some of that knowledge across. [Faculty5] 

According to the lecturers, medical students from Bahrain are confident that transferring background knowledge through translation will guarantee success at university, which results in constructing some sort of an independent practice.

“When I ask the high achievers ‘How do you do it?’, they very quickly tell me they’ve just learnt the material. I think that the high achievers have cracked how they personally learn and they are able to do it. And in most cases they just go through every piece of information we give them and they learn it, whether it’s by rote learning I don’t know, I think that their comprehension issues goes hand in hand with it, they automatically comprehend because they absorb a lot of information, it’s already there. […] They have been able to make that transition almost seamlessly and I often ask them and it seems that what has worked for them in secondary school also works for them here. Obviously they are making some adaptations but they seem to have all the tools that are required to remain high achievers here.” [Faculty2]

Acting on the old strategy in students’ own meaningful ways, as opposed to following the practice given to medical students by the university, was a significant finding in this study. What was also significant is that the medical students were still able to develop this new strategy, despite having the identity that was perceived by the secondary teachers to be inappropriate for higher education. The medical students highlighted this and, for example, one student stated that in school, “they didn’t prepare us at all in terms of studying because they gave us, like, summaries and told us to study those.” (Non-ELC2 Student 3)

Another student also pointed out that “they should teach subjects in English in government schools because when they [students] graduate, they don’t need Arabic skills.” (ELC2 Student 4)

## Discussion

The literature presented at the beginning of this article suggests that when students’ transition to higher education is viewed from the perspective of CoP, this transition is seen as being affected by not knowing the ‘knowing how’ of the university because the new university practices differ from the practices familiar to the students.^22^ By studying Tobbell’s^23^  research, for example, it was argued that exploring transitions using the socio-cultural theory of CoP leads to an understanding of what changes in identity, connected with the move from one educational setting to another, can affect transitions and that these changes are related to having to acquire new skills and developing meaning that are valuable in the new community, but that might not have been valuable in previous settings. 

This study, therefore, began with the assumption that the groups of teachers in national schools in Bahrain and the medical university are two separate communities of practice and that exploring the perceived degree of transferability of schools’ unique practices to the university context would help to understand the transition of the medical students. However, as the research progressed, the emerging themes suggested that an important factor in the transition studied here, perhaps more important than, for instance, the English language attributes formed at secondary level or school pedagogy, was the medical students’ strong feelings about themselves as learners and the strategic actions these students could take, based on the feelings of confidence and empowerment. As suggested in the introduction, CoP in transitions, on the other hand, do not seem to consider the power of learner agency in the new context but rather focus on the power of the new community influencing learners’ identity change.^21^ This has led us to propose that more work related to learner individual agency should be done within CoP as a model for looking at transitions.

CoP position learning as embedded in wider social and cultural practices which are perceived as valuable within a given community.^20^ Therefore, when learners enter a new community, they become peripheral participants whose integration into this community depends on identity shifts they will have to undergo to be able to acquire the practices of the new community.^20^ The way this framework conceptualises transitions suggests that change in identity ‘is in the foreground because the new and strange practices force reconsideration of practice and therefore shifts in identity trajectories’.^21^ This framework therefore proposes that, in transitions, not all practices in one community will cause positive or required identity shifts, which might prevent  participation of students because their contextual practices do not match the practices of the new community of practice.^21^ Taking a similar view, as explained at the beginning of this article, the university where the study was conducted designed a specific orientation programme, in which experienced members of the university community teach new medical students about the practices at the university so they can learn about the required norms as soon as possible.

The findings in this study, on the other hand, suggest that despite the lack of transfer between the contextual practices in schools and the university, participation in the community of the university was reported to still have been achieved by the Bahraini students due to the strong intra-individual agency of the medical students and their strong feelings about themselves as learners. This participation was evidenced by students achieving the learning outcomes, which was the definition of transition adopted in this study. The fact that the medical students knew they could still achieve the learning outcomes using their ‘old ways’ resulted in them being undeterred by the new and strange practices when they became novices on the periphery of the university community. This does not seem to support the central role of these practices in transitions as proposed by CoP^20^^,^^23^ and suggests a greater focus on individual agency within this theory.

The conceptual themes of inadequacy, blame and lack of confidence in school pedagogies and practices related to teaching English and science identified in this research were believed by both groups of school teachers to mark the medical students’ transition. This was expressed in the  interview comments referring to studying in Arabic and then in English as a factor, insufficiency of the intellectual level of the students for transition due to memorisation or experiencing a great shock when at university due to spoon-feeding. This suggested that the learning identities of medical students formerly established in school could be under threat when entering university, as they were expected to find themselves to be puzzled novices who could feel like outsiders and who were unsure about how to progress into the new community. At the same time, the themes in the results section that indicated confidence in background knowledge and study strategies that the medical students already brought with them to the periphery stage, have led us to conclude that those students were able to feel they had become legitimate members of the new community, regardless the perceived inadequacy and the blame on the former school practices.

The findings in this research have therefore suggested that how the medical students felt about their agency – that it was not limited because of the good background knowledge and their ability to transfer learning identities from school without making any major academic adjustments, was more important than the practices that made them successful in school. Believing that the students were fit for medical higher education seemed to enable them to act on their cultural background and to face the challenges in the new community, avoiding in this way problems with transition born out of uncertainty of not knowing ‘the knowing how’ of the university.^8^  O’Donnell and Tobbell^21^ and Leese ^22^ who were cited at the beginning of this article argue that there is no shortcut to gaining membership in communities but through mastery of the required practice and negotiations of identity. The medical students in this study demonstrated that this shortcut exists as, despite being given the practice by the medical university during their orientation period, they decided not to follow it and chose strategies they felt more confident about. 

For instance, in Eckert’s^14^  position on CoP and language development, the use of language within a community and the patterns of language formed in that community are key to legitimate peripheral participation because, as explained at the beginning of this chapter, they are the distinctive characteristics that the members develop. These were perceived unimportant in the transition considered here. Despite the fact that the interview comments suggested that the strategies used to teach English in the communities of Bahraini schools formed patterns that were inadequate for university, the power of the medical students’ agency seem to have compensated for the anxieties connected with the lack of participation at the periphery stage, which could have been caused by inadequate language education in schools.

Additionally, while Wenger^20^ proposes that identity shifts are required by the participation in a new community, the data from this study have suggested that this participation can also depend on what the students already know, how they understand learning and how they package together what they know about learning. The medical students in this study were confident that they could learn by memorisation and their strong feelings about memorisation as a good learning technique allowed them to find ways of applying it to the university context, reinforcing their images of self as learners. So, the practice of the community that was based on memorisation was perceived to be inadequate by  the school teachers but what the medical students did with this practice was inherently proactive and allowed them to preserve the same learner identity from school through to university. The university faculty interviewed in this study also agreed that translating and memorising is not a normal practice at the medical university because the translation process leads to the loss of scientific information. They, however, admitted that students who do it are successful because they achieve the learning outcomes, suggesting at the same time that what Wenger^20^   proposes about identity changes should be further reconsidered in the context of transitions. The data in this research imply that these changes may not be required due to novices’ strong, positive feelings about their study strategies. Wenger^28^ himself argues that boundaries between communities can be crossed by trying to sustain connections between different communities of practice and that learners’ agency gives them the potential to create various forms of continuity between them. Boundary crossing is an important aspect of CoP because, as Wenger ^28^ explains in his later work, ‘at the boundaries, competence and experience tend to diverge (…) [but] when experience and competence are in close tension, (…) learning at boundaries is likely to be maximised’. According to Wenger,^28^ there has to be something at the boundary that causes this tension and one of these things can be an individual’s ability to translate between repertoires. The medical students in this study demonstrated that they are able to translate between the repertoires of schools and the university, supporting what Wenger28  claims about spanning between two communities but perhaps highlighting a bit more the great power of agency in this process.  This was contextualised by the data in this research which suggest that, as opposed to following the new practice that was imposed on them by the university during the orientation week, Bahraini learners chose to sustain the connections between their school and university communities by relying on their old practices and their enhanced sense of agency. This sense of agency seemed to help them form ways of transitioning that spanned boundaries and linked the two communities of practice, but sustaining at the same time their identity as learners throughout the process of transition.

The findings presented in this paper, therefore, seem to imply that more research is needed on the role of students’ agency in the model of CoP in transitions.  Drawing on the criticism by Lea,^29^ and taking into account the findings presented in this article, it can be argued that CoP is a top-down model for understanding transitions which does not seem to take account of what choices students make on the periphery, how they feel about themselves as learners and what they can do with this enhanced feeling to facilitate their transition to higher education.

The findings also problematise the value of CoP as a framework for learning in medical higher education institutions. For example, Jakovljevic, Buskley and Bushney ^30^ propose that CoP are useful because universities which adopt this model to improve the quality of education for international students empower learners through tacit knowledge-sharing opportunities. While these approaches to knowledge-management have been found useful in some instances^21^^,^^22^ the participants in this study indicated that Bahraini learners did not need such opportunities because they were able to work out for themselves how to participate in the new community and they did it in the way that suited them rather than by following the ways recommended by the university. This suggests that today’s medical education needs to take account of the fact that learners exercise a great level of control over structural factors and that they do it in their own, unique ways. This should alert medical educators to the need for shifting away from attempts to institutionalise their systems of learning and putting greater emphasis on prioritising student voices. 

Fox^31^ criticizes CoP for telling us little about how members change old practice into a new one.  Medical institutions that attempt to standardise their teaching through ‘preaching’ of established practices not only do the same but also assume that there is only one way of changing that practice – that is, through acquisition of what they assume is the right way of learning. We argue that such approach to medical education does not capture the question why learners may choose to change the practice differently, or even not to change it at all. Our analysis, therefore, points to the necessity of building cultural competence and the willingness to look into the rationale behind our students’ decisions. 

In terms of limitations of this study, it is worth considering here that Bahraini learners might themselves constitute a community of practice and that the strategies they adopt might only work for them at the beginning of their university careers, or might not be used at all after the first year of study. It seems though that their agency ‘promote[s] specific artefacts to focus future negotiation of meaning in specific ways’^20^ rather than the legitimate practice provided to them by the medical university. These learners have developed their own ways, or what could be called as ‘an independent practice’, to have a satisfying experience at university and in this sense they constitute a community of practice. However, rather than following the practices given to them by the university to achieve the desired goals, they packaged together what they knew about learning to facilitate their transition and this is where the power of their agency is demonstrated.

## Conclusion

The notion of CoP has been influential in contextualising transitions to university by recognising the context in which these transitions take place.^21^^,^^23^  However, this research has suggested that focusing on the educational context in which medical students were located prior to university, and then later during their university years, might not be sufficient to advance our understanding of their success in a medical school. According to CoP, learners beginning higher education are legitimate peripheral participants because they are new to the practices of a university and they will remain on this periphery unless they undergo identity shifts.^20^ Based on what the case study in this paper has suggested, we argued that graduates beginning medical education can sometimes be ‘agentic’ participants – which means that they are able to become legitimate participants in a community of the medical university, not because they change what they know about learning but because they are able to be proactive about their learning identities. Due to the definition of the transition adopted in this study, which was based on changes in identity, the fact that students seem not to shift to the new practices advocated by their new medical school is significant. This has practical implications in that it seems to suggest that transition to university does not necessarily happen through standardised systems of learning in medical schools. This might be different in cases when transition is defined as, for example, developmental change8, because then the fact that students continue to use old practices might mean that they have not made the transition. Adopting this definition of transition can be viewed as another limitation of this study. However, despite the small scale case study design of this research, the findings generate room for new research on agency within CoP as a framework for understanding transitions and place more responsibility on medical institutions to invest into understanding how their students learn.

## Conflict of Interest

The authors declare that they have no conflict of interest.
